# Tracking Perioperative Inflammation over Time: A Prospective Observational Study on the Longitudinal Dynamics of CRP and GDF-15

**DOI:** 10.3390/nu18142255

**Published:** 2026-07-10

**Authors:** Chattarin Pumtako, Donald C. McMillan, Barry J. Laird, Ross D. Dolan, John M. Wadsworth, Donogh Maguire

**Affiliations:** 1 Academic Unit of Surgery, School of Medicine, College of Medical Veterinary & Life Sciences, University of Glasgow, Glasgow G12 8TB, UK; 2 Institute of Genetics and Cancer, University of Edinburgh, Edinburgh EH4 2XU, UK; 3 Scottish Trace Element and Micronutrient Diagnostic and Research Laboratory, Department of Clinical Biochemistry, Macewan Building, Glasgow Royal Infirmary, Glasgow G4 0SF, UK; 4 Emergency Medicine Department, Glasgow Royal Infirmary, Glasgow G4 0SF, UK

**Keywords:** cachexia, GDF-15, ghrelin, systemic inflammation, CRP, appetite regulation

## Abstract

**Background:** C-reactive protein (CRP) is a well-established marker of systemic inflammation, while Growth Differentiation Factor-15 (GDF-15) has emerged as a potential biomarker of cellular stress. Their relative perioperative dynamics remain incompletely defined. This prospective observational study aimed to explicitly characterize and compare the longitudinal dynamics of CRP and GDF-15 in patients undergoing elective knee arthroplasty. Characterizing these biomarkers in a controlled acute surgical model provides clinical relevance by mapping systemic acute-phase inflammation against cellular stress pathways, which may offer a baseline reference for evaluating chronic tissue-wasting states. **Methods:** This prospective observational study included 47 patients undergoing elective knee arthroplasty. After excluding nine patients with elevated baseline CRP (>10 mg/L), serial measurements of CRP and GDF-15 were analysed daily in 38 patients from pre-operation to postoperative Days 1–3. **Results:** CRP rose sharply after surgery, peaking on Day 3 (median: 206.0 mg/L vs. baseline: 3.0 mg/L), representing a maximal increase exceeding 6100%. GDF-15 levels also increased progressively, peaking on Day 3 (median: 1682.5 pg/mL vs. baseline: 968.8 pg/mL), which represented a statistically significant but more modest rise of 33%. CRP and GDF-15 were significantly correlated on postoperative day 1 (rs = 0.53, *p* = 0.001) but not days 2 and 3. Baseline GDF-15, but not CRP, was significantly associated with oral hypoglycaemic agent use and vitamin B12 supplementation. Compared to values reported in recent randomized trials of ghrelin, anti-GDF-15, and anti-IL-6 therapies, GDF-15 levels in this surgical cohort remained lower, while CRP approached levels observed in such studies. **Conclusions:** CRP and GDF-15 rise in parallel following surgical injury; however, GDF-15 shows a considerably lower sensitivity to the inflammatory response. These findings suggest a complementary role of GDF-15 alongside CRP in profiling the perioperative stress response. While its relatively modest acute elevation limits its utility as a primary marker of acute inflammation, this surgical stress model serves as a reference that may help contextualize biomarker profiles in chronic inflammatory or wasting conditions, such as cancer cachexia, rather than serving as a direct tool for cachexia management.

## 1. Background

Systemic inflammation is a hallmark of many chronic diseases and is associated with poor clinical outcomes, including reduced treatment tolerance, impaired physical function, and decreased survival. C-reactive protein (CRP), an acute-phase reactant produced by the liver in response to pro-inflammatory cytokines, is routinely used as a biomarker to assess inflammatory status and prognosis across a range of conditions, including cancer, cardiovascular disease, and chronic organ failure [1]. Elevated CRP reflects an underlying systemic inflammatory response (SIR), which plays a central role in disease progression and metabolic dysfunction, particularly cancer [2,3].

Growth Differentiation Factor-15 (GDF-15), a member of the TGF-β superfamily, is a biomarker of cellular stress, metabolic dysfunction, and disease burden. It is secreted in response to various stressors, including hypoxia, inflammation, and malignancy, and is detectable in the circulation of patients with chronic diseases such as cancer, heart failure, and chronic kidney disease [4].

Elevated circulating levels of GDF-15 have been associated with weight loss, muscle wasting, reduced appetite, and poor overall prognosis in cancer patients [5]. Its mechanistic role in appetite regulation via the GFRAL pathway has generated considerable interest in cachexia research, supporting its potential value as a biomarker of metabolic stress and disease burden [6]. Importantly, although both CRP and GDF-15 have been associated with inflammation-related conditions, they reflect distinct biological domains. CRP primarily represents a classical hepatic acute-phase response, whereas GDF-15 is considered a broader marker of cellular and metabolic stress. Evaluating these biomarkers concurrently within a controlled acute surgical model may therefore provide insight into their relative temporal behaviour and potential complementary biological roles.

Despite growing interest in both biomarkers, few investigations have directly evaluated their relative kinetics within controlled acute surgical models such as elective primary total knee arthroplasty. This model provides a standardized inflammatory stimulus that allows comparison of acute-phase inflammatory and cellular stress responses while minimizing many confounding factors present in chronic disease populations. Understanding these potentially divergent kinetics may help distinguish acute inflammatory responses from broader cellular stress pathways and clarify whether CRP and GDF-15 provide complementary biological information following acute surgical injury. Mechanistically, CRP represents a classical liver-derived acute-phase reactant driven by downstream interleukin-6 signalling, whereas GDF-15 operates across multiple biological domains as a broader, non-specific sensor of intracellular mitochondrial and metabolic stress. Evaluating both biomarkers simultaneously within a controlled surgical environment—such as elective primary total knee arthroplasty—provides an ideal, standardized model to investigate the intersection of immediate, damage-associated molecular pattern (DAMP)-driven acute inflammation against cellular and metabolic stress pathways. Furthermore, from an economic sustainability perspective, while automated assays for GDF-15 are commercially standardized (e.g., Roche Cobas platforms), its current cost remains substantially higher than routine high-sensitivity CRP testing, necessitating a clear definition of its exploratory value before recommendation for routine diagnostic exams.

Therefore, the primary aim of the present study was to examine, in a longitudinal, prospective fashion, the relative values and divergent kinetics of CRP and GDF-15 in a well-characterized cohort of patients undergoing an elective surgical injury. Rather than attempting to directly manage wasting disease, this study serves as an exploratory, hypothesis-generating surgical model to improve our conceptual understanding of how systemic inflammation interacts with homeostatic metabolic reserves, which may provide foundational insights for interpreting biomarker profiles in chronic cachectic conditions.

## 2. Methods

### 2.1. Study Design and Participants

This was a single-centre, non-interventional, prospective observational study involving patients undergoing elective primary total knee arthroplasty for osteoarthritis. The study was registered at ClinicalTrials.gov (NCT03554668) and received ethical approval from the East of England–Cambridgeshire and Hertfordshire Research Ethics Committee (Ref: 17/EE/0270). Eligible participants were adults scheduled for primary knee arthroplasty. To minimize selection bias, recruitment was performed consecutively from the elective surgical waiting list. Exclusion criteria included pre-operative CRP > 10 mg/L, impaired renal function (eGFR < 60 mL/min/1.73 m^2^), and perioperative supplementation with thiamine or magnesium [7]. Patients receiving perioperative thiamine or magnesium supplementation were excluded to minimise potential confounding effects on perioperative biomarker responses, as previously reported in this cohort. These micronutrients have been reported to influence inflammatory and metabolic responses following surgery and could therefore affect the observed kinetics of circulating biomarkers. The rationale for excluding patients with a baseline CRP > 10 mg/L was to isolate the pure acute-phase response triggered by the surgical injury itself and eliminate individuals with pre-existing, active chronic systemic inflammatory conditions. Baseline demographic data, including age, BMI, comorbidities, medication history, and perioperative variables such as anaesthetic technique and fluid management, were recorded. These variables were collected for descriptive purposes and exploratory subgroup analyses examining potential associations with baseline biomarker concentrations.

### 2.2. Sample Collection and Laboratory Measurements

Venous blood samples were collected at four time points: pre-operatively (baseline), and on post-operative Days 1, 2, and 3, between 06:00 and 08:00 after an overnight fast. If a patient was discharged earlier, collection ceased at discharge. Blood samples were processed according to routine hospital laboratory protocols and analysed using accredited automated laboratory platforms as described below. Serum CRP and albumin were measured using an automated analyser (Architect; Abbott Diagnostics, Abbott Park, IL, USA). Neutrophils and lymphocytes were obtained from routine haematological analysis. Plasma GDF-15 levels were determined using an automated analyser (Cobas, Roche Diagnostics, Indianapolis, IN, USA) using the Elecsys GDF-15 V2 enzyme-linked immunosorbent assay (ELISA). According to the manufacturer, the assay has a lower detection limit (analytical sensitivity) of 400 pg/mL with an intra-assay variability (coefficient of variation, CV) of <5%. Blood sampling was consistently conducted at specific daily intervals (exactly 24, 48, and 72 h postoperatively) between 06:00 and 08:00 h following an overnight fast. Serum lactate dehydrogenase (LDH) was measured as part of standard biochemical evaluation. LDH was included as an exploratory marker of tissue injury and cellular stress to provide additional biological context for the perioperative response. It was not considered a primary study endpoint.

### 2.3. Statistical Analysis

All continuous variables were expressed as medians with interquartile ranges (IQR). Data normality was evaluated using the Shapiro–Wilk test for CRP, GDF-15, albumin, LDH, lactate, glucose, white cell count, neutrophil count, lymphocyte count, and platelet count. As these variables demonstrated non-parametric distributions, non-parametric analytical strategies were implemented. Non-parametric analytical strategies were implemented, and methods requiring a normal distribution, such as repeated measures ANOVA, were deemed inappropriate and omitted. Temporal variations in biomarkers across the four distinct time points (pre-op, Day 1, Day 2, and Day 3) were assessed using Friedman’s test for repeated measures. When the global Friedman test indicated significant differences, post hoc pairwise comparisons between specific time points were evaluated using the Wilcoxon signed-rank test. Given the exploratory nature of this observational study, no formal adjustment for multiple comparisons (e.g., Bonferroni correction) was applied. Accordingly, all post hoc findings should be interpreted as exploratory and hypothesis-generating.

Due to the exploratory nature of this observational study, a formal a priori sample size calculation was not executed; however, the cohort size of 38 analysed patients was deemed justified based on historical standards in previous perioperative biomarker dynamics literature utilizing this reproducible model. For longitudinal data tracking and correlation analyses, missing observations arising from early patient discharge before Day 3 were handled via available-case analysis utilizing pairwise deletion, ensuring all available valid pairs were utilized for each specific temporal correlation. Spearman’s rank correlation coefficients (rs) were used to examine relationships between biomarkers at each specific time point. A *p*-value < 0.05 was considered statistically significant, and all analyses were conducted using SPSS software (version 29.0, IBM Corp., Armonk, NY, USA).

## 3. Results

Forty-seven patients, who underwent elective primary knee arthroplasty due to osteoarthritis, were initially enrolled in the study. Of these, nine patients (19%) exhibited preoperative evidence of systemic inflammation, defined as CRP > 10 mg/L, and were therefore excluded from the final analysis (Table 1). The remaining cohort consisted of 38 patients. The median age was 66 years (IQR 58–73) and the median BMI was 32.5 kg/m^2^ (IQR 28.7–38.5). The majority of participants were over 65 years of age (63%), female (53%), and classified as obese (68%) (Table 1). Exploratory subgroup analyses suggested possible associations between baseline GDF-15 concentrations and oral hypoglycaemic agent use (n = 2) as well as vitamin B12 supplementation (n = 4). Given the extremely small subgroup sizes, these observations should be interpreted with considerable caution and regarded as hypothesis-generating only. No significant associations were identified between clinicopathological variables and baseline CRP concentrations.

**Figure 1:**
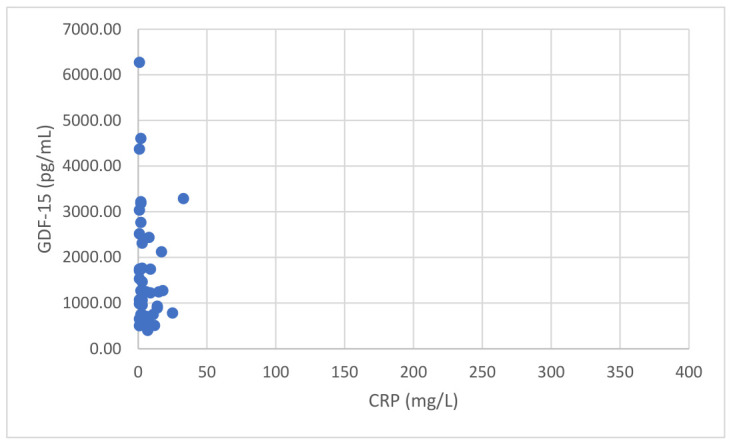
Preop CRP vs. GDF-15 Spearman r = −0.231, *p* = 0.136.

**Figure 2:**
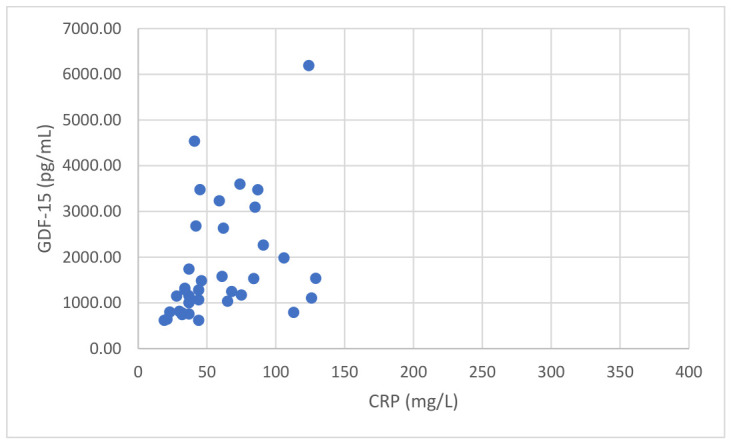
Day 1 CRP vs. GDF-15 Spearman r = 0.525, *p* = 0.001.

**Figure 3:**
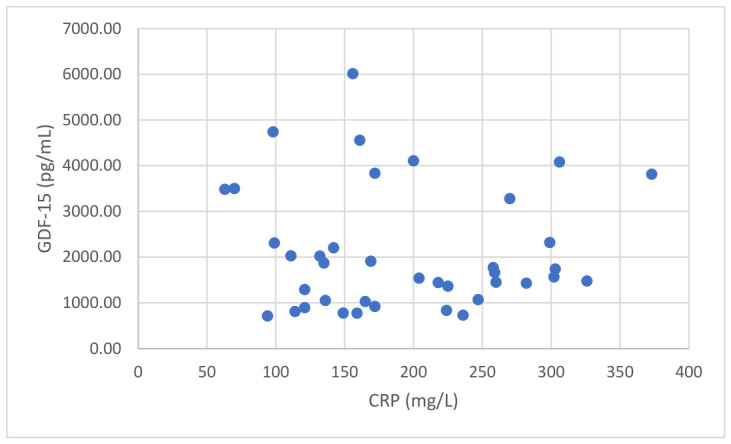
Day 2 CRP vs. GDF-15 Spearman r = 0.024, *p* = 0.883.

**Figure 4:**
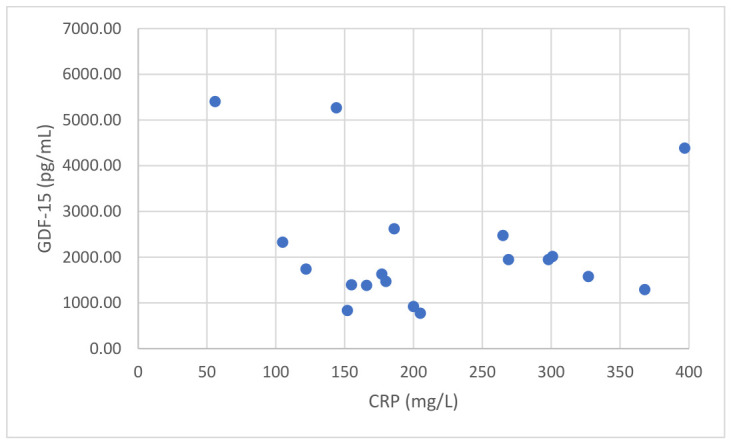
Day 3 CRP vs. GDF-15 Spearman r = 0.111, *p* = 0.652.

Scatter plots of CRP and GDF-15 levels across the perioperative period revealed a dynamic relationship between these two biomarkers of systemic inflammation and stress response. At baseline, between pre-operative CRP and GDF-15 concentrations (Figure 1), there was no significant correlation (rs = −0.231, *p* = 0.136), with most patients exhibiting low CRP and a wide range of GDF-15 concentrations. Following surgery, however, the relationship became more apparent. On post-operative Day 1 (Figure 2), both CRP and GDF-15 levels began to rise, with an emerging significant positive association (rs = 0.525, *p* = 0.001). However, this association was not sustained on postoperative Day 2 (Figure 3) (rs = 0.024, *p* = 0.883) or postoperative Day 3 (Figure 4) (rs = 0.111, *p* = 0.652). Thus, the only statistically significant correlation between CRP and GDF-15 was observed on postoperative Day 1.

**Figure 5:**
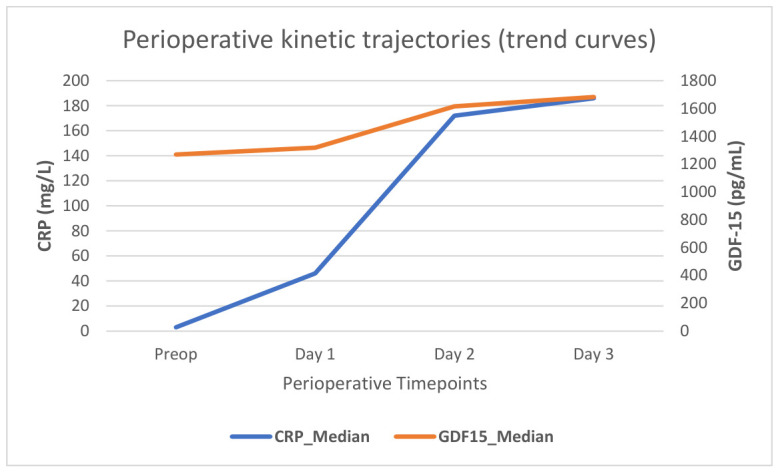
Perioperative kinetic trajectories of circulating CRP and GDF-15 across the four evaluation time points. Continuous lines represent median values (with CRP plotted on the primary left *Y*-axis and GDF-15 plotted on the secondary right *Y*-axis), demonstrating a synchronous but independent magnitude of elevation in response to acute surgical stress.

Table 2 summarizes the perioperative fluctuations in key circulating biomarkers following elective knee arthroplasty. Median values with corresponding interquartile ranges (IQR) [25th–75th percentiles] are reported at four time points: preoperatively, and on postoperative days 1, 2, and 3. The maximal percentage change from baseline (%Δ) and corresponding *p*-values (Friedman test) are also presented. Among the evaluated biomarkers, CRP demonstrated the most dramatic acute-phase response, increasing progressively from a baseline median of 3.0 mg/L to 186.0 mg/L by Day 3, representing a maximal increase of 6100% (*p* < 0.001). This substantial mathematical magnitude of the maximal percentage change in CRP (+6100%) is primarily driven by the remarkably low, sterile baseline median concentration (3.0 mg/L) observed in this healthy elective arthroplasty cohort preoperatively, reflecting a robust and typical acute-phase systemic inflammatory response to major surgical trauma. Glucose, Neutrophil count, and lactate increased sharply on Day 1 representing a maximal increase of 42%, 130%, 80%, respectively (*p* < 0.001). In contrast, lymphocyte and platelet counts fell, representing maximal decreases of 29% (*p* < 0.001) and 16% (*p* < 0.001), respectively. Albumin concentrations fell progressively to day 3, representing a maximal decrease of 25% (*p* < 0.001). GDF-15 concentrations increased progressively to Day 3, reaching a peak median of 1683 pg/mL and representing a maximal increase of 33% (*p* < 0.001). Notably, LDH concentrations also showed significant temporal variation over the study period, peaking on Day 1 with a 19% maximal increase (*p* = 0.029). To better conceptualize the temporal relationship between the two primary biomarkers, a dual-*Y*-axis trajectory model was constructed (Figure 5). This visual presentation clearly illustrates the synchronous, time-dependent escalation of both circulating CRP and GDF-15, highlighting a parallel progression in their median kinetic paths from the preoperative baseline through to postoperative Day 3.

To further characterize the relationship between acute-phase inflammation, metabolic cellular stress, and nutritional status, correlation analyses were performed among serum albumin, CRP, and GDF-15 across the perioperative period. At baseline, no statistically significant correlations were observed among the three biomarkers (all *p* > 0.05 $). On postoperative Day 1, a significant positive correlation emerged between circulating CRP and GDF-15 concentrations (rs = 0.525, *p* = 0.001), coupled with a marginal inverse trend between GDF-15 and serum albumin levels (rs = −0.315, *p* = 0.054). However, this coordinated response was transient, as no statistically significant or consistent correlations were sustained among serum albumin, CRP, or GDF-15 on postoperative Days 2 and 3 (all *p* > 0.05).

## 4. Discussion

These findings suggest that GDF-15 rises in parallel with CRP following elective knee arthroplasty and may reflect a complementary dimension of the post-surgical inflammatory stress response. Peaking on postoperative Days 2–3. CRP, a well-established acute-phase reactant, demonstrated a marked elevation (up to ~6100% from baseline), reflecting its rapid hepatic synthesis in response to IL-6 and tissue injury [8]. GDF-15, by contrast, showed a more modest increase (~30–80%). This magnitude of change is consistent with the expected cellular stress response associated with major elective surgery and remains substantially lower than concentrations typically reported in chronic wasting conditions. While pre-operative CRP and GDF-15 were only modestly correlated, their association became more apparent postoperatively, indicating co-regulation during systemic inflammation. These findings show that GDF-15 responds to surgical stress, albeit with a considerably lower sensitivity than CRP.

CRP is widely used to detect systemic inflammation and one such clinical application is in its use in the Global Leadership Initiative on Malnutrition (GLIM) criteria for the definition of cachexia [9]. Recently, GDF-15 has emerged as a promising biomarker for cachexia due to its role in appetite suppression via the GFRAL pathway and its relationship with weight loss and poor prognosis [10,11,12]. Given the present results, it remains to be established whether GDF-15 has a role in the GLIM criteria in patients with cancer [13]. Although CRP and GDF-15 may reflect different biological domains, the present findings are insufficient to determine whether combined biomarker profiling provides additional clinical value beyond established inflammatory markers. Further studies in chronic disease populations are required before any role within cachexia phenotyping frameworks can be considered.

The comparison between CRP and GDF-15 responses to surgical stress may be influenced by differences in their biological kinetics and clearance rates, making direct quantitative comparisons challenging. GDF-15 is known to accumulate more slowly and persist longer than classical acute-phase proteins like CRP. Nevertheless, in the present study GDF-15 concentrations were consistent with previous studies showing GDF-15 as a slower, sustained stress-responsive cytokine [14,15]. In clinical samples, GDF-15 levels often exceed 3000 pg/mL at baseline. For example, in the recent Phase 2 trial of ponsegromab (anti-GDF-15 antibody), cancer patients prior to treatment, had median GDF-15 levels over 3200 pg/mL. In the present study, using the same assay, median GDF-15 levels peaked below this threshold (~2300 pg/mL), suggesting that surgical stress alone may not induce the level of metabolic dysregulation seen in cancer cachexia. The lack of sustained correlation between GDF-15 and albumin on Days 2–3 further indicates that after the initial surgical hit, GDF-15 kinetics diverge from traditional hepatic acute-phase markers.

Interestingly, the perioperative behaviour of GDF-15 observed in our cohort mirrors findings reported by Skipworth et al. [13], who demonstrated that circulating GDF-15 levels were significantly elevated in patients with cancer cachexia (compared with controls), particularly those with systemic inflammation and weight loss. While their study focused on a chronic cachectic setting, our data reveal that even acute surgical stress is sufficient to induce a measurable rise in GDF-15, albeit of a smaller magnitude. This supports the concept that GDF-15 is not solely a biomarker of malignancy-associated wasting but also a broader indicator of systemic metabolic stress. This interpretation is supported by recent work demonstrating that simple clinical variables can accurately estimate appendicular skeletal muscle mass (ASM), highlighting the importance of muscle status as a component of metabolic health and physiological resilience. Although body composition was not assessed in the present study, future investigations incorporating direct measures of muscle mass may help further clarify the relationship between GDF-15, metabolic stress, and functional status [16].

A key limitation of the present study is the short observational window, which was restricted to the first three postoperative days. Consequently, the long-term kinetics and full resolution profile of GDF-15 cannot be definitively established without extended follow-up points (e.g., Days 7, 14, or 30). During this acute timeframe, the biomarker profiles are likely dominated by rapid, immediate post-surgical cytokine fluxes and damage-associated molecular pattern (DAMP)-driven innate immune activation. While CRP concentrations at Days 2–3 remain elevated due to well-characterized hepatic production kinetics, the rising trajectory of GDF-15 during this specific window might reflect ongoing tissue cellular stress and subsequent repair mechanisms rather than a true chronic metabolic plateau. Future longitudinal studies with extended follow-up are required to map the long-term tracking of GDF-15.

Furthermore, the significant baseline associations identified between GDF-15 concentrations and both oral hypoglycaemic agents (n = 2) and vitamin B12 supplementation (n = 4) must be interpreted with extreme caution. These subgroup analyses involve very small numbers of patients, rendering the statistical comparisons highly susceptible to Type I errors and statistical instability. These observed relationships are likely confounded by unmeasured clinical variables, including advanced age, underlying variations in renal function, diabetes severity, or specific supplementation regimens, and warrant validation in larger, dedicated cohorts.

In summary, despite some limitations, the present study provides important insights into the perioperative behaviour of GDF-15 and highlights its distinct profile compared to traditional inflammatory markers such as CRP. These findings may help contextualise GDF-15 responses observed in chronic inflammatory and tissue-wasting conditions.

## Figures and Tables

**Table 1:** The relationship between baseline CRP, GDF-15, and clinicopathological characteristics of patients who underwent elective surgery for knee arthroplasty (n = 47). Note: Subgroup statistical analyses involving clinical variables with extremely small sample sizes (e.g., oral hypoglycaemic agents [n = 2] and insulin use [n = 1]) were executed without formal adjustment for multiple comparisons. These exploratory *p*-values are highly susceptible to Type I error and statistical instability; therefore, these specific baseline associations must be interpreted with extreme caution and treated strictly as preliminary, hypothesis-generating observations.

Variable		n	CRP Median (Min–Max)	*p*-Value	GDF-15 Median (Min–Max)	*p*-Value
Age > 65 years	Yes	27	3.0 (1.0–33.0)	0.109	1709 (623–6274)	<0.001
	No	20	6.0 (1.0–18.0)		893 (400–2122)	
Sex (Female)	F	26	3.0 (1.0–18.0)	0.164	983 (538–4372)	0.358
	M	21	2.0 (1.0–33.0)		1532 (400–6274)	
BMI > 30	Yes	33	3.0 (1.0–25.0)	0.178	1223 (400–4605)	0.060
	No	14	3.0 (1.0–33.0)		1764 (587–6274)	
CRP > 10	Yes	9	15.0 (11.0–33.0)	-	1087 (750–3288)	0.455
	No	38	2.0 (1.0–9.0)		1257 (400–6274)	
Smoker	Yes	8	3.0 (1.0–25.0)	0.526	1369 (782–4372)	0.497
	No	39	3.0 (1.0–33.0)		1243 (400–6274)	
PPi	Yes	27	4.5 (1.0–33.0)	0.157	1234 (400–4605)	0.439
	No	20	2.0 (1.0–15.0)		1256 (502–6274)	
OHA	Yes	2	1.5 (1.0–2.0)	0.206	3560 (2516–4605)	0.046
	No	45	3.0 (1.0–33.0)		1233 (400–6274)	
Insulin	Yes	1	3.0 (3.0–3.0)	0.935	1284 (1284–1284)	0.883
	No	46	3.0 (1.0–33.0)		1257 (400–6274)	
Diuretics	Yes	8	1.5 (1.0–7.0)	0.062	2102 (400–6274)	0.173
	No	39	4.5 (1.0–33.0)		1244 (502–4605)	
CCB	Yes	18	7.0 (1.0–25.0)	0.170	1355 (400–6274)	0.930
	No	29	3.0 (1.0–33.0)		1114 (502–4605)	
ACEi	Yes	9	12.0 (1.0–25.0)	0.537	1233 (782–1532)	0.449
	No	38	3.0 (1.0–33.0)		1257 (400–6274)	
Statin	Yes	23	3.0 (1.0–33.0)	0.312	1467 (502–6274)	0.575
	No	24	7.0 (1.0–18.0)		930 (400–4372)	
MTX	Yes	4	9.0 (1.0–17.0)	0.915	2122 (1742–4372)	0.381
	No	43	3.0 (1.0–33.0)		1223 (400–6274)	
Sulphazalazine	Yes	2	6.0 (3.0–9.0)	0.464	2027 (1742–2312)	0.292
	No	45	3.0 (1.0–33.0)		1243 (400–6274)	
Steroids	Yes	4	9.0 (1.0–17.0)	0.885	1827 (1532–2122)	0.118
	No	43	3.0 (1.0–33.0)		1233 (400–6274)	
Vitamin D	Yes	8	2.5 (1.0–17.0)	0.401	1620 (538–4372)	0.610
	No	39	4.5 (1.0–33.0)		1233 (400–6274)	
Vitamin B12	Yes	4	1.5 (1.0–3.0)	0.107	3796 (1467–6274)	0.009
	No	43	4.5 (1.0–33.0)		1103 (400–4605)	
Folic acid	Yes	3	9.0 (1.0–17.0)	0.477	3247 (2122–4372)	0.439
	No	44	3.0 (1.0–33.0)		1233 (400–6274)	

**Table 2:** Longitudinal Profiles of Circulating Biomarkers Following Elective Knee Arthroplasty.

Variable	Median (Percentile25–Percentile75)				% Δ	*p*-Value
	Preop	Day 1	Day 2	Day 3		
Glucose	5.4 (5.1–6.0)	7.7 (6.8–9.1)	6.6 (6.2–7.3)	6.4 (5.9–7.3)	42	<0.001
LDH	214.0 (179.5–233.5)	255.0 (211.0–276.0)	217.0 (204.8–295.0)	231.0 (192.5–273.0)	19	0.029
Lactate	1.0 (0.9–1.4)	1.8 (1.3–2.3)	1.4 (1.1–2.0)	1.2 (0.9–1.6)	80	<0.001
WCC	6.9 (6.0–7.6)	12.3 (10.9–13.9)	11.0 (9.1–12.5)	8.6 (7.4–10.6)	78	<0.001
Neutrophils	4.3 (3.6–5.0)	9.9 (8.5–11.1)	8.2 (6.8–9.7)	6.6 (5.1–7.7)	130	<0.001
Lymphocytes	1.7 (1.2–2.3)	1.2 (1.0–1.7)	1.3 (0.9–1.8)	1.3 (0.9–1.7)	−29	<0.001
Platelet	245 (227–295)	225 (203–275)	204 (185–252)	204 (173–243)	−16	<0.001
CRP	3.0 (1.0–9.0)	46.0 (37.0–85.0)	172.0 (132.8–255.3)	186.0 (154.3–292.0)	6100	<0.001
Albumin	40.0 (37.0–42.0)	35.0 (34.0–37.0)	34.0 (32.0–35.0)	30.0 (29.0–33.3)	−25	<0.001
GDF15	1269 (750–2122)	1318 (1003–2634)	1615 (1033–3039)	1683 (1312–2436)	33	<0.001

## Data Availability

The datasets generated and/or analysed during the current study are available from the author on reasonable request due to restrictions privacy reasons.
